# Regenerative Medicine for Equine Musculoskeletal Diseases

**DOI:** 10.3390/ani11010234

**Published:** 2021-01-19

**Authors:** Iris Ribitsch, Gil Lola Oreff, Florien Jenner

**Affiliations:** Equine Surgery Unit, Department of Companion Animals and Horses, University of Veterinary Medicine Vienna, Veterinaerplatz 1, 1210 Vienna, Austria; iris.ribitsch@vetmeduni.ac.at (I.R.); gil.oreff@vetmeduni.ac.at (G.L.O.)

**Keywords:** regenerative medicine, musculoskeletal, equine, horse, stem cell, platelet-rich plasma, autologous conditioned serum, orthopaedic

## Abstract

**Simple Summary:**

Lameness due to musculoskeletal disease is the most common diagnosis in equine veterinary practice. Many of these orthopaedic disorders are chronic problems, for which no clinically satisfactory treatment exists. Thus, high hopes are pinned on regenerative medicine, which aims to replace or regenerate cells, tissues, or organs to restore or establish normal function. Some regenerative medicine therapies have already made their way into equine clinical practice mainly to treat tendon injures, tendinopathies, cartilage injuries and degenerative joint disorders with promising but diverse results. This review summarises the current knowledge of commonly used regenerative medicine treatments and critically discusses their use.

**Abstract:**

Musculoskeletal injuries and chronic degenerative diseases commonly affect both athletic and sedentary horses and can entail the end of their athletic careers. The ensuing repair processes frequently do not yield fully functional regeneration of the injured tissues but biomechanically inferior scar or replacement tissue, causing high reinjury rates, degenerative disease progression and chronic morbidity. Regenerative medicine is an emerging, rapidly evolving branch of translational medicine that aims to replace or regenerate cells, tissues, or organs to restore or establish normal function. It includes tissue engineering but also cell-based and cell-free stimulation of endogenous self-repair mechanisms. Some regenerative medicine therapies have made their way into equine clinical practice mainly to treat tendon injures, tendinopathies, cartilage injuries and degenerative joint disorders with promising results. However, the qualitative and quantitative spatiotemporal requirements for specific bioactive factors to trigger tissue regeneration in the injury response are still unknown, and consequently, therapeutic approaches and treatment results are diverse. To exploit the full potential of this burgeoning field of medicine, further research will be required and is ongoing. This review summarises the current knowledge of commonly used regenerative medicine treatments in equine patients and critically discusses their use.

## 1. Introduction

### 1.1. Equine Musculoskeletal Disease: Clinical Need and Burden of Disease 

Lameness due to musculoskeletal disease is the most common diagnosis in equine veterinary practice [1,2]. Correspondingly, orthopaedic problems are the primary cause of loss of use and death in athletic horses [3,4,5,6,7,8,9], causing more than 70% of days-lost to training in both show jumpers and racehorses [4,5,8]. While the prevalence of lameness increases with age, reaching 51% in horses aged 15 years and older and 77% in geriatric horses above 30 years [4,10,11], even in a cohort of 4–5-year-old horses, 24% showed moderate to severe orthopaedic clinical findings at a standard riding horse quality test [3,4,12].

The type and anatomical location of the musculoskeletal problems differ between athletic disciplines, competition levels and age [2,5,13,14]. Although the causes of lameness in horses competing at low levels of dressage and show jumping are very similar to each other and those of pleasure horses, different injury predispositions emerge in the various sports with increasing level of competition and athletic demands, placed upon the horse [13,14]. Generally, articular and tendon/ligament disorders, due to their insufficient healing capacity and the consequent tendency to develop chronic disorders, have by far the greatest clinical relevance in most disciplines [4]. The superficial digital flexor tendon (SDFT) is commonly injured in racing, elite eventing and show jumping and distal deep digital flexor tendon (DDFT) in elite show jumping [14]. Dressage horses are at a higher risk of hindlimb and racehorses of forelimb suspensory desmitis [14]. Additionally, foot pain and degenerative joint disease (= osteoarthritis, OA) of the distal tarsal joints are frequent clinical findings in sport horses and barrel racers [13,15], while in Thoroughbred racehorses stress fractures, carpal and metacarpo-/metatarsophalangeal joint injuries are a significant cause of morbidity [4,7,16,17]. In aged horses, OA and chronic laminitis are the most common disorders [4]. The importance of musculoskeletal disease in equine practice is emphasised by lameness being the principal reason for the euthanasia of geriatric horses [4,5,18]. Additionally, previous musculoskeletal problems almost doubled the incidence-rate ratio of training days-lost due to orthopaedic injury, further highlighting the clinical demand for regenerative treatments [4,5,18].

### 1.2. Regenerative Medicine Overview: Development of the Field, First Successes, Challenges Preventing Wide-Spread Implementation 

**Figure 1:**
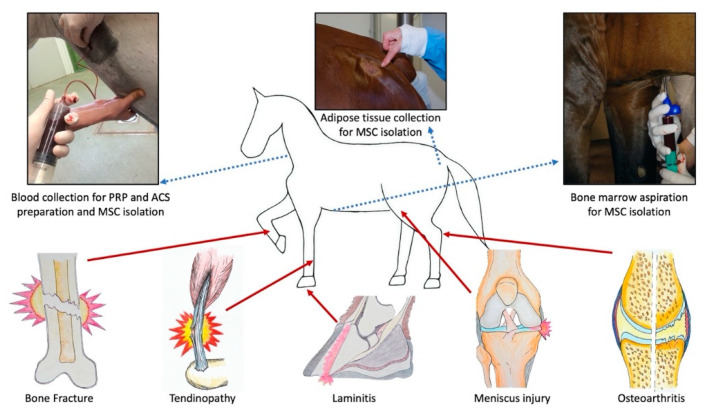
Illustration of the equine musculoskeletal diseases discussed in this review and the harvest sites for bone marrow (from the sternum), adipose tissue (from the tail head) and blood (from the jugular vein) for mesenchymal stem cell (MSC) isolation, respectively, for platelet-rich plasma (PRP) and autologous conditioned serum (ACS) preparation.

Regenerative medicine (RM) is an emerging, rapidly evolving branch of translational medicine that aims to replace or regenerate cells, tissues, or organs to restore or establish normal function lost due to disease, damage, age, or congenital defects [19,20]. RM is a broad field that includes tissue engineering (TE) but also cell-based and cell-free stimulation of endogenous self-repair mechanisms in organs and tissues. In equine practice, several regenerative therapies, such as mesenchymal stem cells (MSCs), platelet-rich plasma (PRP), autologous conditioned serum (ACS) and autologous protein solution (APS), have entered clinical use for various musculoskeletal indications over the last decade (Figure 1). However, the field of RM still has to live up to high hopes and expectations placed on it, both from a medical and financial viewpoint.

Although promising results were achieved in multiple experimental and preclinical studies, case reports and even first small randomised and controlled studies, large placebo-controlled studies are still scarce [21,22,23]. Furthermore, the field of RM faces several challenges like the lack of well-defined cells to be used as therapeutics and insufficient understanding of their mode of action. 

To exploit the full potential of tissues to heal, our understanding of how reparative processes are mediated and may be directed towards regeneration rather than scarring repair needs to be improved. Currently, the mechanisms of the tightly regulated process, involving the interplay of growth factors, cytokines, proteinases, and cellular mediators combined with differences in cellular density, proliferation rate, inflammatory response, extracellular matrix (ECM) composition and synthetic function, are still poorly understood [24,25,26].

Only the answers to the questions arising from these challenges will allow the field to gain well-founded evidence-based results—putting years of preclinical and in vitro experience onto a basis which will pave the way for large scale and routine clinical applications. 

The field of equine regenerative medicine involves much pioneering work with variable treatment protocols using different routes of administration and/or dosages of cells respectively bioactive factors, which may contribute to the discrepancies between promising experimental in vitro as well as in vivo results and clinical effectiveness [27]. Hence, intensive research efforts are still ongoing and required to find ways to exploit the maximal potential of RM. As the field is still in its infancy and rapidly evolving, this review also includes in vitro studies and basic science papers as well as case reports as indicators of new developments and possibilities in equine RM. The detailed information of all referenced in vivo studies conducted in horses or using equine cells is summarised in Supplementary Materials Table S1. 

#### 1.2.1. Mesenchymal Stem Cells

One major tool of RM are mesenchymal stem cells (also known as multipotent mesenchymal stromal cells, multipotent stromal cells, medicinal signalling cells, MSCs). MSCs are defined as plastic adherent cells with the ability to differentiate into osteoblasts, adipocytes and chondroblasts in vitro, which express a characteristic panel of markers, including CD105, CD73 and CD90, while lacking expression of surface molecules specific to other cell types [28]. While bone marrow and adipose-derived stem cells are the best-researched stem cell sources of humans and animals, perinatal sources such as cord blood, umbilical cord tissue, amniotic membranes, or amniotic fluid are also commonly used [29,30,31,32,33,34,35,36,37,38,39,40,41,42,43,44,45,46,47,48,49,50]. 

MSCs show different intrinsic properties depending on their tissue of origin as well as donor age [29]. To further our understanding of their therapeutic potential and optimise their clinical application, it is essential to study the properties and specificities of MSCs derived from different sources. This is exemplified by the higher proliferation rate, longer lifespan, and lower immunogenicity of juvenile MSCs derived from perinatal tissues compared to cells from adult donors [33,51,52,53,54]. Additionally, juvenile MSCs have a broader differentiation capability towards cell types of endo- or ectodermal origin [33,46].

Initially, isolated and culture-expanded MSCs were thought to regenerate tissue via engraftment and differentiation [55]. However, since the survival and engraftment of MSCs in the target tissue following transplantation are negligible, mounting evidence suggests that MSCs exert their therapeutic effect predominantly by secreting bioactive factors (the “secretome”) that modulate the immune response, reduce inflammation, inhibit cell death, and induce and stimulate endogenous regeneration [56,57,58,59,60,61,62,63]. While already many clinical trials exploring the use of MSCs for the treatment of a wide variety of diseases are ongoing, their intrinsic properties and mechanism of action, as well as the role of their microenvironment in modulating their behaviour and function are not yet fully understood and require further study to achieve their full therapeutic potential [57,62,64,65,66].

#### 1.2.2. Autologous Blood Products

In addition, autologous blood products, the effect of which is based on the secretome of blood cells, are employed in regenerative medicine. Autologous blood products are minimally manipulated medicinal products, comprising plasma- or serum-based blood derivates, obtained from the patient’s own blood. Based on their contents, different products with different properties are distinguished. The best-researched and hence clinically most frequently applied products are PRP and ACS. Both PRP and ACS aim at reducing inflammation, protecting intact and newly formed tissue, recruiting cells such as MSCs, macrophages, and other pro-regenerative cells and at supporting neovascularisation by supplying growth factors, cytokines, and nutrients. Autologous blood products are used clinically to treat tendon, ligament, cartilage, and bone pathologies [64]. However, the composition of these products may vary considerably depending on inter- and intraindividual factors (physiologic state of the patient, status of the immune system, day time, time of year, etc.) and the sample processing technique (centrifugation time and force, number of centrifugations, activation, incubation, etc.) [65,66,67], impeding comparison of study results. Therefore, product and study standardisation are significant research challenges, which need to be overcome to achieve reliable therapeutic outcomes.

PRP is derived from the liquid phase of blood through centrifugation to increase the platelet concentration. It is defined as a volume of plasma with a platelet count greater than whole blood [68]. As compared to ACS, it is obtained from anticoagulated blood without incubation. The therapeutic effect of PRP is mainly caused by degranulation of the platelets’ alpha-granules, which leads to the release of a plethora of growth factors and cytokines, including platelet-derived growth factor (PDGF), insulin-like growth factor (IGF), transforming growth factor-beta (TGF- β1), vascular endothelial growth factor (VEGF), fibroblast growth factor (FGF), and platelet-derived epidermal growth factor [68,69].

The relevance of absolute platelet concentrations for the treatment effect is yet uncertain. The current recommendation was extrapolated from human medicine and suggests that a viable platelet concentrate should contain 3–5× baseline platelets [68]. However, substantiating evidence is scarce. Similarly, the role of leukocytes (white blood cells, WBCs) in PRP is discussed controversially. On the one hand, neutrophils release proinflammatory cytokines, matrix metalloproteinases (MMPs) and reactive oxygen species which may exacerbate the disease or disorder to be treated [68,69,70,71]. On the other hand, WBCs increase the concentration of growth factors in PRP and, may hence elicit a beneficial effect [72]. Currently, there is no conclusive evidence indicating that white cells should be either included or depleted from PRP.

ACS is a cell-free product obtained from the liquid phase of blood after coagulation (coagulation takes place during a defined conditioning/incubation phase) and centrifugation, which is free of coagulation factors such as prothrombin and fibrinogen but contains globulins and albumin. During coagulation/incubation with borosilicate beads, platelets and white blood cells in the blood sample are activated to release growths factors and cytokines [73]. The spectrum of released factors is similar to PRP, but the concentration is different because, in contrast to PRP, platelets are not enriched. The therapeutic effect of ACS may further be related to higher levels of IL-1Ra (Interleukin 1 Receptor Antagonist Protein) and IGF which are thought to be central players inhibiting the destructive cytokine cascade in degenerative joint disease [64,74,75]. However, incubation time and conditions may have a considerable influence on ACS cytokine and growth factor content and concentration and should, therefore, be carefully evaluated [76].

In contrast to PRP, ACS is injected three times, with a widely used treatment interval of one week, but the treatment intervals vary between different studies [77].

Finally, ACS has one practical advantage over PRP: ACS is cell-free and can hence easily be frozen and stored as compared to PRP. However, recently first reports describing possible storage conditions for PRP were released [78,79].

## 2. Regenerative Therapies by Disease Area

### 2.1. Tendon/Ligament

#### 2.1.1. Clinical Need and Burden of Disease

Pathologic changes in tendons due to repetitive use and overstrain, with exercise and ageing as significant contributing factors, are amongst the most frequently occurring musculoskeletal problems in sport and pleasure horses [80,81] and the leading cause of injury during racing [82]. The equine superficial digital flexor tendon (SDFT) is the structure most at risk for suffering an injury with a tendinitis incidence of up to 43% [83,84,85]. In racehorses, SDFT disease accounts for 89–90% of all tendon and ligament injuries, the remainder being suspensory ligament injuries [16,86].

Due to the equine quadruped-specific anatomy characterised by the proximally located muscles and the distally located tendons in combination with the hyper-extended metacarpophalangeal joint, equine tendons are exposed to enormous forces during an athletic workout. Maximal strains in the SDFT range around 16%, which is close to the functional limit, during galloping in Thoroughbreds [84,87]. In addition to positioning the limb during locomotion, the SDFT functions like a spring, storing energy during the stance and releasing it during the swing phase. Only intact molecular composition and organisation enable tendons to fulfil these requirements.

Equine tendon healing processes are traditionally classified into three distinct, temporally coordinated but overlapping phases: the acute inflammatory phase, which begins immediately following injury and lasts only a few days is followed by the subacute reparative or proliferative phase, which peaks at 3–6 weeks and the chronic remodelling phase, which can last for 12 months after injury [81,82,83,88]. However, the repair of adult tendons is slow and inefficient due to the low cellularity, vascularity and metabolic rate of the tendon and commonly associated with persistent, non-resolving inflammation, yielding a fibrovascular scar with significantly inferior biomechanical properties [81,88,89]. Growing evidence supports the contribution of inflammation to the development of tendinopathy [90]. Although the posttraumatic inflammatory response is an integral component of the healing response and is required for debridement following injury, persistent inflammation may be related to dysregulated degradation and deposition of ECM components and contribute to the inadequate regenerative capacity of tendons by driving fibrosis [81,88,90,91,92,93]. The resulting fibrovascular scar is characterised by a disorganised matrix structure and increased production of proteoglycans, glycosaminoglycans and collagen type III [81,88,90,91,92,93]. Differences in biomechanical characteristics due to changes in the structure and molecular composition of the tendon matrix inevitably result in impaired tendon function. Therefore, even after an apparent initial recovery, reinjury rates of up to 80% and chronic morbidity are reported [6,29,83,94]. The 10-fold increased risk to sustain SDFT injuries in the 60 days following veterinary examinations for a tendon problem further confirms the impact of pre-existing tendon pathologies [95]. As a result, a considerable number of equine patients, especially racing Thoroughbreds but also event, dressage and show jumping horses, are forced to end their sporting career early due to tendon injuries [6,80,85].

A controlled exercise program alone or in combination with a variety of conservative treatments, such as corrective shoeing and nonsteroidal anti-inflammatory drugs (NSAIDs), is still the gold-standard therapy for equine tendon disease. However, no current treatment can restore the functional properties of injured tendons. To improve tendon healing, new treatment strategies, aiming at full restoration of the tendon functionality, need to be developed, which is only possible if regeneration ad integrum can be achieved. Successful treatment is likely to require modulation of inflammation and promotion of proresolution processes with disease-stage specific therapeutic interventions.

#### 2.1.2. Regenerative Therapies

Since the first report suggesting bone marrow-derived MSCs (bmMSCs) for intralesional tendon injection as potential new therapy for injuries of the equine SDFT [29], MSC therapies have been shown to significantly decrease reinjury rates from 80% [83] to 13–36% [94] and to achieve a more tendon like repair tissue with better histologic architecture and biomechanical properties of the healed tendon tissue compared to traditional treatments [87,93,94,96].

However, the mechanisms accounting for the beneficial therapeutic effect are still not fully understood. What is known so far is, that following MSC transplantation, decreased infiltration of immune cells into the injured tissue, a reduction in proinflammatory cytokine concentration and an increased expression of anti-inflammatory cytokines is observed [97,98]. Additionally, MSCs were reported to inhibit the TGF-β1 signalling pathway, a driving force in fibrosis development. Furthermore, it was shown that autologous MSCs enhance perfusion and neovascularisation of the healing tendon tissue [99]. In summary, MSCs are thought to improve the balance between synthesis and degradation of the ECM and to reduce fibrosis as reviewed by Usunier et al. [97].

Initially, to treat tendon lesions, MSCs were exclusively applied intralesionally. More recently, it was shown that autologous MSCs applied intravenously, or intra-arterially, may also elicit a beneficial effect in the treatment of tendinopathies when compared with anti-inflammatory drugs [30,100,101,102].

However, there are also studies which show that the effect of a single intralesional treatment with autologous MSCs may be limited [22,103]. Therefore, some studies have investigated the effects of combining MSCs with PRP or other blood-products or tenogenically differentiating MSCs for tendon repair to improve clinical outcome and prolong therapeutic effects [104,105,106]. Additionally, conditioned medium obtained from amniotic membrane progenitor cells has been investigated, based on the notion that the MSC treatment effect is mainly based on their paracrine activity [107].

To broaden the spectrum of stem cell sources available for clinical applications and reduce time to injection, allogeneic MSC applications moved into the focus of interest. Lack of MHC II in MSCs was proven in multiple studies and administration of allogeneic cells in most cases did not result in an adverse or inflammatory reaction that would compromise their use [108]. Thus, MSCs are considered safe for allogeneic administration [109], which has opened the way for the application of allogeneic MSCs derived from cord blood, cord tissue or amnion to utilise the potentially higher regenerative capacities of juvenile cells [35,106,107,110].

Over the past decades, PRP has become a common treatment for tendon injuries and suspensory ligament desmitis. PRP contains specific growths factors, such as PDGF, IGF, TGF- β1, VEGF and FGF, which play important roles in tendon and ligament healing [69]. Results of an in vitro study suggest that the beneficial effect of platelet-rich gel supernatants may lie in mediating the release of anti-inflammatory cytokines, inhibiting IL-1β, and increasing release of IL-4, IL-1Ra and PDGF [111].

Several studies have shown promising effects leading to improved neovascularisation, better organisation of the collagen network and higher strength of the regenerated tissue, after treatment with PRP as compared to controls [21,64,112,113,114,115]. In one of the rare prospective, randomised controlled trials Geburek et al. showed that a single intralesional treatment with PRP could contribute to an earlier reduction of lameness and an advanced organisation of repair tissue compared to saline treatment. In particular, it was demonstrated that the fibrillar matrix is getting organised into fascicles [21].

In racehorses, a beneficial effect on rehabilitation time, numbers of horses returning to racing and numbers of races entered after PRP treatment compared to controls was reported [116,117].

However, in several in vitro as well as in vivo studies, in which the effect of PRP was compared to other treatments, PRP did not always lead to the best outcome. When comparing PRP treatment to extracorporeal shock wave therapy, both RRP and EST lead to positive results [23]. Horses with more severe ultrasound changes responded better to PRP, but more horses treated with shock wave therapy returned to work [23]. The comparison of bone marrow and adipose tissue-derived MSCs to PRP revealed clear positive effects of all treatments compared with the controls. However, bmMSCs resulted in a better outcome than PRP and adipose-derived MSCs [38]. Similarly, in an in vitro study comparing the gene expression patterns and DNA content of suspensory ligament explants, it was concluded that acellular bone marrow might be preferable over PRP as a blood-based biological for suspensory ligament tissue regeneration based on its more stable stimulation of decorin and COMP expression [113]. While PRP may have an anabolic effect on matrix synthesis by suspensory ligament fibroblasts, the effect was even greater with acellular bone marrow [112].

However, despite all these studies, comparison of therapeutic efficacy remains tricky because of the different study protocols and PRP preparation techniques used [64]. Nonetheless, initial results are promising and warrant further investigation using standardised study and PRP preparation protocols.

The efficacy of ACS in treating equine naturally occurring tendinopathies was also evaluated, although it is predominantly used for the treatment of osteoarthritis [118]. The results of the study demonstrated an early significant reduction of lameness and temporary improvement of ultrasonographic morphology of the repair tissue. Furthermore, ACS treatment decreased proliferation and increased ECM productivity of tenocyte as demonstrated by elevated collagen type I expression [118].

### 2.2. Osteoarthritis

#### 2.2.1. Clinical Need and Burden of Disease

Osteoarthritis (OA) is the primary cause of lameness and thus of disability to perform in horses [4,119]. Indeed, approximately 60% of equine lameness is related to OA [119,120]. In horses older than 15 years, the prevalence of OA is greater than 50%, and in horses over 30 years, it increases to 80–90% [4,11,121].

**Figure 2:**
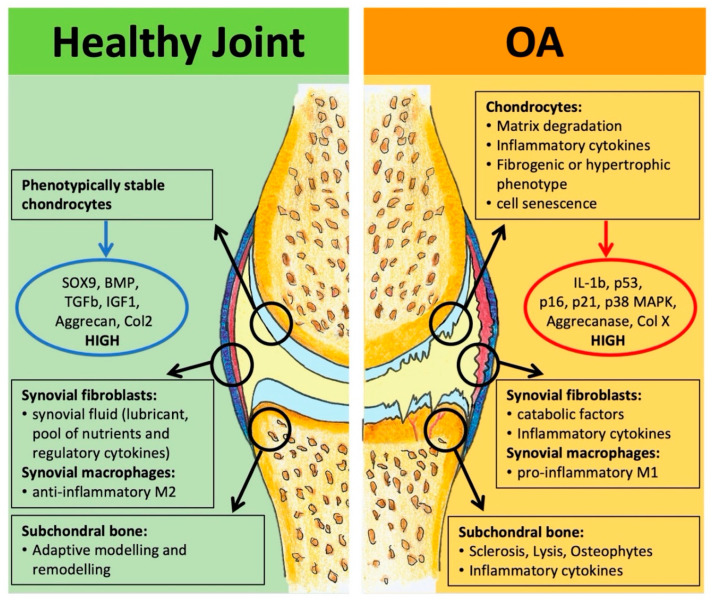
Characteristics of the different articular cells and tissues in healthy joints and in osteoarthritis (OA).

OA is a complex, multifaceted disorder, which is characterised by cartilage degeneration, inflammation, (premature) cartilage ageing, chondrocyte senescence and phenotypic transitions (dedifferentiation and hypertrophic differentiation of chondrocytes). However, it is a disease of the entire joint, affecting all articular tissues because of their physical and functional association [122]. OA may occur as a result of a variety of predisposing factors such as age, mechanical injury, genetics, gender, metabolic dysfunction and obesity that incite a cascade of pathophysiological events within articular tissues [123,124]. Irrespective of the initiating factor(s), the pathogenesis of OA follows a common molecular pathway, which is orchestrated by intricate crosstalk between chondrocytes, synovial macrophages and fibroblasts, osteocytes and osteoblasts and infiltrating leukocytes as well as the ECM of articular tissues and synovial fluid (Figure 2) [124,125,126,127,128,129,130,131,132,133]. OA manifests in cartilage degradation, fibrillation and mineralisation, loss of type II collagen and proteoglycans, increased chondrocyte synthetic activity, proliferation and apoptosis, synovial inflammation, hyperplasia and hypertrophy, subchondral sclerosis, and osteophyte formation [123,134].

Chondrocytes in physiologic adult articular cartilage are phenotypically stable, maturationally arrested, differentiated cells that maintain tissue homeostasis by synthesising a very low level of ECM to replace damaged matrix molecules, thereby preserving the structural integrity of the cartilage matrix [135]. OA is associated with the loss of constraints that maintain the correct chondrocyte phenotype, the physiologically tightly regulated low turnover of the ECM of articular cartilage and the functionality of central homeostatic mechanisms [136]. The normally quiescent chondrocytes undergo a phenotypic shift in response to injury and become activated, characterised by cell proliferation, cluster formation and increased production of both extracellular matrix proteins and matrix-degrading enzymes [137]. One of the most striking features of OA is the high phenotypic pleomorphism and substantial heterogeneity in gene expression patterns and cellular responses displayed by osteoarthritic chondrocytes in contrast to their physiological counterparts [135]. Many of the biological changes in osteoarthritic chondrocytes mimic the differentiation patterns in foetal skeletogenesis [135]. In particular, hypertrophic differentiation of chondrocytes is normal during the development of cartilage and endochondral bone and appears to be aberrant in OA [135,138].

The pathophysiological events are driven principally by an early innate immune response that progressively catalyses degenerative changes. Much of the innate immune activation and cytokine production in the OA joint is attributed to synovial proinflammatory macrophages, the key effectors of synovial inflammation, that show significantly growing numbers with increasing grade of inflammation, but fibroblast-like synoviocytes and chondrocytes also substantially contribute to OA pathogenesis [139,140,141,142,143,144,145]. Cartilage ECM degradation products released into the synovial microenvironment further stimulate the production of catabolic and proinflammatory mediators and proteolytic enzymes, creating a vicious cycle of cartilage breakdown and synovial inflammation [136,145,146].

In addition to contributing to cartilage breakdown, the inflamed synovium has a significant role in the osteoclastogenesis of subchondral bone in OA. Subchondral bone is a source of inflammatory mediators implicated in clinical OA pain, hypertrophic differentiation of chondrocytes and the degradation of the deep layer of cartilage and is involved in the abnormal distribution of stress on the bone–cartilage interface secondary to sclerosis and remodelling of the subchondral bone [124,147,148,149]. The interplay between damaged articular tissues and infiltrating immune cells contributes to chronic inflammation, the loss of cellular homeostasis, an imbalance between matrix synthesis and degradation and thus disease progression [132,138,150].

Exposure to inflammatory and oxidative mediators also enhances premature stress-induced senescence and ageing of chondrocytes resulting in an accumulation of senescent cells in the superficial layer of the articular cartilage and the synovium in OA, which in turn secrete a variety of inflammatory cytokines and matrix-degrading proteases known as the senescence-associated secretory phenotype (SASP) [151]. The SASP influences cell plasticity and propagates senescence and inflammation to surrounding cells and tissues, contributing to the degenerative microenvironment of OA. Cellular senescence, inflammation and metabolic abnormalities driven by OA-associated risk factors are accompanied by epigenetic modifications, which also have an essential role in regulating chondrocyte hypertrophy and catabolic processes.

Adult articular cartilage has very limited ability for self-repair and current treatment strategies, such as NSAIDs and intra-articular injections with corticosteroids, are only palliative in nature and have little impact on the progressive degeneration of articular cartilage [152,153,154,155,156]. Consequently, there is a large unmet need for efficacious disease-modifying therapies, and thus a growing interest in regenerative medicine approaches.

#### 2.2.2. Regenerative Therapies

Currently, debridement and marrow stimulation techniques are still the main techniques used to treat equine cartilage defects. These techniques are simple and cost-effective but do not lead to regeneration of articular cartilage [157]. Additionally, autologous chondrocyte implantation (ACI) and matrix-induced autologous chondrocyte implantation (MACI), which were shown to improve clinical outcome, do not result in restoration of hyaline cartilage with equivalent biomechanical properties as the native tissue [157,158,159,160]. Furthermore, the complexity of these procedures and the high costs, are serious drawbacks for routine applicability in equine surgery [157]. There is thus an ongoing search for novel techniques that would sustainably restore the form and function of articular cartilage.

MSCs are considered a promising cell type for cartilage repair [55]. Preclinical and clinical studies in rats, goats and humans have demonstrated the potential of MSCs to improve joint function and patient’s osteoarthritis index (WOMAC, Western Ontario and McMaster Universities Osteoarthritis Index), to reduce pain and to decrease the size of cartilage lesions following intra-articular injection [161,162,163]. Furthermore, they may support hyaline regeneration by modulating joint homeostasis [63,164]. However, several studies in horses showed that despite the improvement of clinical symptoms and histologic appearance of the cartilage repair tissue [164,165,166,167], MSC therapies in the long-term still do not achieve regeneration of hyaline cartilage, but yield inferior fibrocartilaginous repair tissue at the defect site [157]. This may in part be due to the limited survival and engraftment of MSCs and the harsh biomechanical and inflammatory environment of the osteoarthritic equine joint [157].

To further improve the effect of MSC therapies proinflammatory or chondrogenic priming strategies for MSC prior to injection were tested, the latter of which was proven to be safe although differentiation of equine bmMSCs may increase the expression of immunogenic proteins [31,168,169]. Additionally, the intra-articular administration of allogeneic and even xenogeneic MSCS for the treatment of OA has been tested in multiple clinical studies in horses with equivocal results [156,170,171]. Although some studies show an immune reaction to allogeneic and xenogeneic stem cells [172], the immune response in vivo seems to be mild, and allogeneic MSCs application has been reported to be safe for intra-articular use in equine patients [173,174,175,176].

To eventually reach the goal of hyaline cartilage regeneration, further well-designed, prospective, randomised controlled and standardised in vitro as well as in vivo trials will be required to compare novel technologies to current ‘gold standard’ clinical approaches [157].

Due to its reported anabolic and anticatabolic effects on articular chondrocytes, PRP became a promising treatment option for OA [177,178]. Despite multiple caveats regarding the intra-articular administration of PRP, which are mainly based on its proinflammatory potential due to its leukocyte content, complement activation capability and the fact that PRP coagulates following injection, it is nowadays increasingly being used and studied in the context of OA [179,180,181,182,183,184,185,186,187,188,189,190,191,192,193,194]. Most clinical studies performed in horses reported a beneficial effect after an initial transient exacerbation of joint inflammation which seems to have no long-term deleterious effects on joint homeostasis [182,195]. This was also confirmed by PRP injections into healthy equine joints [196,197], but the safety of intra-articular PRP administration in general and different PRP preparations in particular is still controversially discussed. For example, it was shown that thrombin activation prior to application could cause increased joint effusion and periarticular signs of inflammation indicating that thrombin activation may not be recommended for PRP for intra-articular application [196]. In vitro results further indicate that the anti-inflammatory and anabolic effects of the platelet products depend on the concentration and the cellular and molecular profile of the PRP-derived product used as well [178,181].

In vivo results are also not uniformly positive but show a beneficial trend in most equine studies. One study demonstrated significant improvement in lameness grade, range of motion and gait kinetics after PRP injection into OA joints [198]. In another study, in which platelet lysate was used to treat OA of the distal interphalangeal joint nine out of 10 horses returned to full athletic use. As expected, no significant radiographic improvements and hence no full joint regeneration was observed, and horses gradually returned to their initial degree of lameness [199]. In contrast, PRP treatment did not lead to statistically significant gait improvement in horses with moderate to severe forelimb OA, [200].

In summary, the effects of platelet products vary greatly based on the research model and cellular content of the platelet product [181].

Similarly, the results of several studies evaluating the efficacy of ACS for treating OA have been equivocal [75], which may be due to the considerable interindividual variability of cytokine- and growth factor content of ACS and the fast clearance from the synovial fluid after intra-articular injection [74,75,201]. On the one hand, ACS has been shown to significantly alleviate clinical symptoms of OA in horses and improve histologic findings compared with placebo controls [202]. On the other hand, in a clinical trial including 19 horses, 11 responded to treatment, whereas eight did not [74].

The therapeutic benefit of ACS may be related to its high levels of IL-1Ra [74,75,202]. Interleukin 1 beta (IL-1β) is a major driver in the development and progression of OA. Therefore, antagonising IL-1β seems to be an obvious strategy to treat OA and slow down disease progression [203]. Nonetheless, the concomitant elevation of other factors suggests that these cytokines may play an essential role in clinical improvements as well [73].

However, in ACS similar to PRP not only anti-inflammatory (IL-1Ra, TGF-β, IL-10) but also proinflammatory cytokines (IL-1β, IL-6, TNF-α, and OSM), in particular TNF-α, may be increased and concentrations of contained cytokines and growth factors may vary greatly [73,77]. For clinicians, it is particularly important to consider that surgical stress may influence the cytokine content of ACS [73,204].

Nonetheless, the results of in vitro and in vivo trials using ACS for the treatment of OA are promising and indicate a potential disease-modifying and anti-inflammatory effect. Treatment with ACS caused upregulation of IL-10 expression in synovium and of type II collagen and aggrecan expression in cartilage explants. In contrast, PGE_2_ concentrations were significantly reduced following treatment with ACS [205]. In a recent in vivo study, a long-time beneficial effect of ACS applied to osteoarthritic horses at two-day intervals was shown, based on synovial fluid IL-1Ra, IL-1 β, C12C, CP II, and CS 846 concentrations [77].

In this context, it is essential to emphasise, that, in contrast to PRP, ACS is obtained from the liquid phase of the blood after coagulation and that hence the caveats associated with the fibrin content of PRP and the coagulation post-injection do not apply for ACS.

### 2.3. Meniscus

#### 2.3.1. Clinical Need and Burden of Disease

The lateral and medial menisci are C shaped wedges of fibrocartilage located between the femur and the tibia in the stifle joint [206,207]. Both contain a thicker convex portion at the peripheral edge and a thinner concave part towards the central edge, a concave femoral surface, and a flat tibial surface. The menisci have ligamentous attachments to the femur, tibia, joint capsule, and one another [206,208]. There are several crucial functions of the menisci such as stabilisation of the joint, increasing joint congruity, assisting with joint lubrication, shock absorption, load transmission and stress reduction [206,207].

Lameness originating from the stifle joint is relatively common in horses, and meniscal injury is one of the primary sources of pain in this joint. In two studies, the incidence of meniscal injury was as high as 34% [209] and 68% [210] of horses with stifle injury that underwent arthroscopy. Meniscal injury in horses is frequently diagnosed with concurrent pathologies such as soft tissue damage, osteoarthritis, and cartilage defects and less often with subchondral bone cysts [208,209,210,211,212,213]. The most common meniscal injury is tearing of the cranial horn of the medial meniscus and its associated cranial meniscotibial ligament. Although those tears are frequently diagnosed with other lesions, they are commonly recognised as the main cause of lameness [209,210,212,213,214,215]. Initiation of the tear takes place usually at the cranial meniscotibial ligament and extends longitudinally further into the cranial horn. Due to this characteristic appearance, a grading system was created with a higher grade involving greater separation of tissues and further extension into the cranial horn [206,207,208,212,213,214]. Meniscal injury in horses, unlike in other species such as humans and dogs, rarely involves injury of the cruciate and collateral ligaments and the pathophysiology behind it is still mostly unknown [212,213]. Few studies were conducted in recent years for better understanding of meniscal tear pathogenesis. It was shown that the cranial meniscotibial ligament appears to be comprised of two units, which become more visually distinct as the stifle is extended. During hyperextension of the stifle joint in horses, the abaxial component of the ligament faces very high tensile forces that may place this region at greater risk of injury [215]. Additionally, since the cranial horn is firmly attached to the tibia, during extension, significant translocation and deformation occur at this region [216].

Diagnosis of soft tissue injuries in the stifle joint can be challenging due to the size and location of the joint, and the unspecific clinical signs. As a result, diagnosis is usually based on a combination of imaging modalities such as radiography, ultrasonography and magnetic resonance imaging (MRI) [212,216,217,218]. The gold-standard treatment for diagnosed meniscal tears is endoscopy for partial meniscectomy and debridement of debris [209,210,212,213]. The configuration of the joint in horses and the inaccessibility of the menisci make it almost impossible to apply other repair techniques as used in human medicine such as suturing or replacements [212]. Conservative treatment including rest, pain and anti-inflammatory drugs and intra-articular medications is also available, although outcome assessment is lacking in current data [212].

The prognosis for return to athletic use depends on the severity of the disease (grade of the tear) and the presence of concurrent pathologies but is overall considered guarded at best. In the few case series that assessed outcome in stifles’ soft tissue injuries, return to activity was noted in approximately 40% of cases [209,210,212,213]. Due to the relatively low success rates of current therapies, regenerative approaches have gained more interest in recent years and achieved some encouraging results [178,219,220,221,222].

#### 2.3.2. Regenerative Therapies

An in vivo study reporting regeneration of meniscal tissue following local delivery of adult MSCs to injured joints in a caprine model of OA [219], raised high hopes for the regeneration of injured meniscus tissue, which were further encouraged by several in vitro studies for equine meniscal cells and explants [178,220,221,222].

However, to date, progress is very limited, and no tissue engineering approaches are available for equine patients suffering from meniscus injuries. The complex structure of the meniscus’ ECM, the heterogeneous cell shapes and properties and the rigorous biomechanical loading menisci are exposed to in vivo, constitute major challenges for meniscus tissue engineering. Ideally, a meniscus implant should resemble the size, shape, vascularity, and biomechanical properties of a natural meniscus to comply with the load-distributing demands in the knee. Available meniscus implants (exclusively for application in humans) use allograft menisci, autologous tissues such as perichondrium or tendon, natural scaffold materials such as fibrin, hyaluronan and collagen, or synthetic materials such as polycaprolactone-urethane, Teflon, or polyurethane [223,224]. Natural materials have also been combined with cell seeding [225]. Besides the obvious lack of vascularisation, current implants do not sufficiently reflect the microstructure and anisotropic tensile properties of native menisci. Their common limitations include inferior load-bearing properties and non-physiologic distribution of loads in the knee, leading to controversial clinical results [225,226,227,228]. The different biomechanical properties of these implants compared to native tissue highlight the need for cellular ingrowth and formation of ECM to gain sufficient biomechanical properties [227,228,229]. The 3D environment is an essential feature of engineered meniscus constructs to facilitate a stable fibrochondrogenic phenotype and increased fibrochondrogenesis. An attempt to address these challenges for the equine field was made by Kremer et al., who cocultured meniscal cells and MSCs in collagen type I hydrogel on a small intestinal matrix [221]. Unfortunately, the developed construct does not yet meet the biomechanical resilience to replace native meniscus tissue adequately and lacks vascular supply.

In another study, equine meniscus sections were reapposed with fibrin glue or fibrin glue plus equine bmMSCs and implanted subcutaneously into nude mice [230]. After harvesting, constructs with bmMSCs showed significantly increased vascularisation, subjectively decreased thickness of the developed repair tissue and increased bonding of the meniscus sections as compared to fibrin alone [230].

However, despite promising in vitro and in vivo results, equine meniscus regeneration is not yet ready for clinical implementation, and several challenges, including implantation of a tissue-engineered meniscus construct into an equine knee, still need to be overcome.

The treatment of confined meniscus defects might be comparatively more straightforward. Investigations into the in vivo regenerative capacity of a collagen scaffold seeded with MSCs which was implanted into a standardised equine meniscus defect showed fibrocartilaginous regeneration 12 months after treatment as compared to the controls which were only partially or not repaired at all [231].

### 2.4. Bone

#### 2.4.1. Clinical Need and Burden of Disease

Bone physiology, pathology, and repair play an important role in equine medicine, mainly due to the challenges of fracture repair. Although other applications, such as the treatment of bone cysts [232,233], have gained some interest in recent years, most of the equine bone research is focused on fracture repair [234,235,236,237,238,239,240,241,242,243].

Fractures in horses are difficult to treat and often lead to euthanasia either due to the primary injury or complications that follow. Furthermore, equine fractures and their treatments involve significant expenses and hold great economic interest, especially in racehorses [239,242,244,245,246,247]. The incidence of fractures in racehorses can be as high as 1–2% per race start, and it might involve either the front or the hind limbs [246,247,248]. Fractures in racehorses are the most common severe musculoskeletal injury, accounting for up to 74% of fatal injuries during racing [17,18,19]. Many studies were conducted to try and recognise risk factors for horses’ fatalities during racing, in the attempt to develop prevention strategies and to improve animal welfare and riders’ safety [16,243,246,249,250,251,252].

Due to their heavy weight and the requirement for rapid return to full weight-bearing on all four limbs to prevent the development of support limb laminitis, fracture repair and management is more challenging in the horse than most other species. Complications of osteosynthesis are common in equine patients and include infection, instability, contralateral limb laminitis, angular limb deformities and cast sores [234]. Additionally, refracture during recovery from surgery is a major concern [234]. Therefore, case selection for osteosynthesis should be done carefully and consider many criteria such as horse characteristics (weight, age, temperament), fracture configuration, available equipment, and costs [253].

Numerous studies were conducted to improve treatment options and strategies for fractures and to reduce complications [234,242,244,245,254,255,256,257,258,259,260,261,262,263]. However, despite advances in surgical technique, implant design, antimicrobial delivery, and perioperative care [234,254,255,256,260,261,262,263,264,265,266,267,268,269], the difficulties facing equine fracture patients are still manifold. As a result, considerable interest has been shown in recent years regarding new biological approaches for bone healing.

#### 2.4.2. Regenerative Therapies

Due to the urgent clinical need, a variety of regenerative approaches have been developed to accelerate osteogenesis. Bone healing is a complicated, tightly orchestrated process with three overlapping phases, each characterised by different cellular and molecular features and extracellular matrix components: an inflammatory phase, a reparative phase (also named the callus formation phase) and a remodelling phase. Immediately following trauma, a hematoma forms at the injury site and an inflammatory response is elicited, initiating the influx of immune and mesenchymal progenitor cells and the release of bioactive factors essential for angiogenesis and bone repair. During the remodelling phase, first a fibrocartilaginous and then a bony callus is formed providing more stability and vascularisation. In the final phase of fracture healing, excess callus is resorbed, and the normal bone architecture restored [235,236,237,262,270,271,272]. New therapeutic approaches are based on and utilise some of the cells, growth factors, cytokines and signalling molecules involved in bone regeneration [235,236,237,239,273,274,275,276]. For example, bone morphogenetic protein-2 (BMP2), which plays essential roles in cell differentiation and osteoinduction, has been used successfully to enhance bone healing [235,236,237,241,272,273].

Recruitment of MSCs from the bone marrow, and other progenitor cells originating from the periosteum or endosteum, is crucial for bone healing. At the fracture bed, those cells can differentiate to chondroblasts or osteoblasts, which will give rise to either chondrocytes or osteocytes, the main cells in cartilage and bone, respectively. Due to their substantial contribution to bone healing, it is not surprising that most regenerative therapy strategies for bone healing are based on stem cells, with or without the support of different growth factors or scaffolds. MSCs can be isolated from different sources and used in their progenitor form or after differentiation [235,236,237,275]. It is still unclear if the source and differentiation pattern of the cells have a significant effect on bone healing, mostly since the field is still lacking in clinical studies.

Nevertheless, research in the field reveals different results regarding stem cell capacities in bone healing and the importance of their source and differentiation. MSCs isolated from bone marrow were shown to have better osteogenic properties than other sources [41]. The same osteogenic capacity was noted in equine induced pluripotent stem cells (iPSCs) [277]. In another study, equine bmMSCs injected with fibrin glue in a murine model, were able to support bone formation much better than MSCs originated from muscle or bmMSCs injected without the scaffold. All cells in this work underwent osteogenic differentiation prior to injection [278]. Less encouraging results were shown in a study using ostectomy of the 4th metacarpal bone as a fracture model. In this study, injection of osteoprogenitor cells with or without fibrin glue did not differ from the control in bone healing [275]. It is interesting to note that the cell source in this study was the periosteum. However, since no other cell sources were used, a comparison between the therapeutic potential of cells from different origins is not possible.

To date, autologous bone grafting is still considering the gold standard when dealing with equine fractures. Although providing the three key criteria for bone regeneration (osteogenesis, osteoinduction and osteoconduction), donor site morbidity is a major limitation of bone grafting [270,279]. Thus, other grafting techniques are being tested as carriers for cells and factors or/and as osteoconductive materials. The most common ones in use are fibrin glue, gelatin, collagen and calcium/tricalcium phosphate ceramics [279]. In a study by Perrier et al., the use of calcium phosphate cement with BMP2 in a fracture model in horses showed superior results in bone healing compared to the control group or even the autologous cancellous bone graft group [241]. Similar promising results could be seen in a study by Seo et al., which examined the use of gelatin/β-tricalcium phosphate sponges with different concentrations of bmMSCs and BMP2 [274]. The combination of the sponges with the cells and BMP demonstrated good bone healing compared to sponges alone or sponges with cells but without BMP. Best results were evident when the BMP concentration was high, regardless of the cell amount [280]. With the increasing availability of 3D printers that enable easier handling and mixing of material in different architectural designs, bone replacements are becoming more and more innovative in recent years [238,240]. These advances hold promise for the future possibility to design replacements based on the fracture configuration with a wide variety of materials.

As platelets have an essential role in fracture healing by contributing to the hematoma formation, and later on by releasing growth factors from their alpha-granules, the potential of PRP to promote bone regeneration seems evident [270]. In a meta-analysis of PRP in the animal long bone model by Gianakos et al., which included 29 studies, 89% reported improved bone healing with PRP shown in histology and 100% in radiographs [281]. Administration of PRP in a donkey suffering from delayed healing of a tibia fracture also reportedly promoted healing within a few weeks following injection [282]. Although clinical research in the field of PRP (and regenerative therapies in general) for bone repair is limited, promising results could be seen from in vitro and experimental in vivo trials, which lay the base for further research in the future.

### 2.5. Laminitis

#### 2.5.1. Clinical Need and Burden of Disease

Laminitis is a disease characterised by inflammation or disruption of the lamellae located inside the hoof [283,284,285,286,287,288]. The epidermal and dermal lamellae connect the hoof wall and the distal phalanx (aka pedal bone) and thus have a crucial role in maintaining the normal suspensory apparatus of the hoof. While the epidermal lamellae are non-sensitive and avascular, the dermal lamellae have a vast vascular network in a thick matrix of connective tissue [288]. Damage to the supporting lamellae will disrupt the delicate balance of the supporting mechanism, resulting in separation of the pedal bone from the hoof wall. In severe cases, this separation can lead to penetration of the bone through the sole of the hoof or complete detachment of the hoof from the underlying bone [283,285,287,288].

Laminitis can affect more than one limb at a time and is a highly painful disease, posing a significant threat to all Equidae. Since weight-bearing on all four limbs is essential for horses, the degree of pain and damage caused by laminitis may require euthanasia in many cases.

The prevalence of the disease can be high and ranges between 1.5% and 34%. The high range of incidence is related to the difference in breeds, location, management and more [286,289,290,291]. Laminitis can occur due to any number of systemic or local insults and appear as an acute or chronic form [283,284,287,292,293,294]. The most common and known aetiologies for laminitis include endocrinopathies, sepsis/endotoxemia and overloading (supporting limb laminitis due to injury of the contralateral limb) [283,284,285,287,292,293,294,295,296,297,298,299].

The prognosis for laminitis depends on the initiating cause and is generally favourable to poor [290,291,300]. Current treatment options are mainly limited to pain management, cryotherapy, hoof support and, depending on the aetiology, treating the underlying disease [287,289,290,291,301,302]. Since no curative treatment is available, high hopes are pinned on new regenerative treatment strategies.

#### 2.5.2. Regenerative Therapies

In both acute and chronic forms of laminitis, an inflammatory response occurs at the lamellae leading to endothelial cell swelling, leukocyte infiltration, oedema and the production of proinflammatory cytokines such as IL-1 β and IL-6 [283,303].

Based on MSCs’ regulatory and immunomodulatory capacities [304,305], it was proposed to use MSCs for laminitis treatment in the attempt to regulate the severity of the inflammatory response in the hoof [306]. In a study by Angelone et al., nine horses with chronic laminitis were injected three times with MSCs suspended in PRP through the palmar digital veins [306]. All horses were treated previously with conventional laminitis treatments without much success. Both allogeneic and autologous MSCs were used without any complications. In the long term, a significant improvement could be noted in vascularity, structure, and function of the hoof [306]. It should be noted that the distribution of MSCs injected to the distal limb might be improved by using different injection methods, such as intra-arterial rather than intravenous injection and thus may improve the therapeutic efficacy [307].

As PRP is a product which contains high levels of growth factors and anti-inflammatory factors, it can aid in regulating inflammation, decreasing pain and assist with angiogenesis. Due to those abilities, it was proposed as a therapeutic option for chronic laminitis [308,309]. Although the literature reporting treatment of laminitis with PRP is limited to case reports, the results are encouraging. Chronic laminitis patients reportedly showed improvement in comfort levels and hoof conformation following injection of PRP through the coronary band and into the hoof [308,309].

Regenerative therapies in the field of laminitis are gaining more interest in recent years, and although the numbers are still small, more studies attempting to improve the welfare of laminitic horses can be expected.

## 3. Future Perspectives

### 3.1. Regulatory

The regulatory framework of regenerative medicine is complex, and many veterinarians are not aware of the implications. Nonetheless, it is important to know that cell products are considered animal drugs and as such their production and application is regulated by the European Medicines Agency (EMA) and the United States Food and Drug Administration (FDA) as well as national regulatory authorities.

The production of MSC based animal drugs is tightly regulated. For other cell-based therapeutics like autologous blood products regulations are less strict because they are widely considered minimally manipulated medicinal products comprising blood cells and/or their products obtained from the patient’s own blood.

Some regulatory aspects may, however, differ between different countries. In Germany, for example, treating veterinarians are allowed to produce cell-based therapeutics like MSCs in their own practice and administer them to their patients under their responsibility [310]. This does also apply for PRP and ACS [310].

In the UK, equine stem cell centres are authorised by the Medicines Directorate (VMD) for the production, processing, and storage of equine stem cells for the autologous treatment of non-food-producing horses.

However, most of the applied regenerative therapies are still at an experimental state and patients are treated within the scope of clinical trials. Yet, it should be mentioned that only recently the first stem cell-based veterinary product using peripheral blood-derived MSCs which were preconditioned with TGF-β1 towards the chondrogenic lineage received market authorisation for the treatment of mild to moderate lameness due to joint inflammation in horses [168,311].

### 3.2. Novel Regenerative Therapies

**Figure 3:**
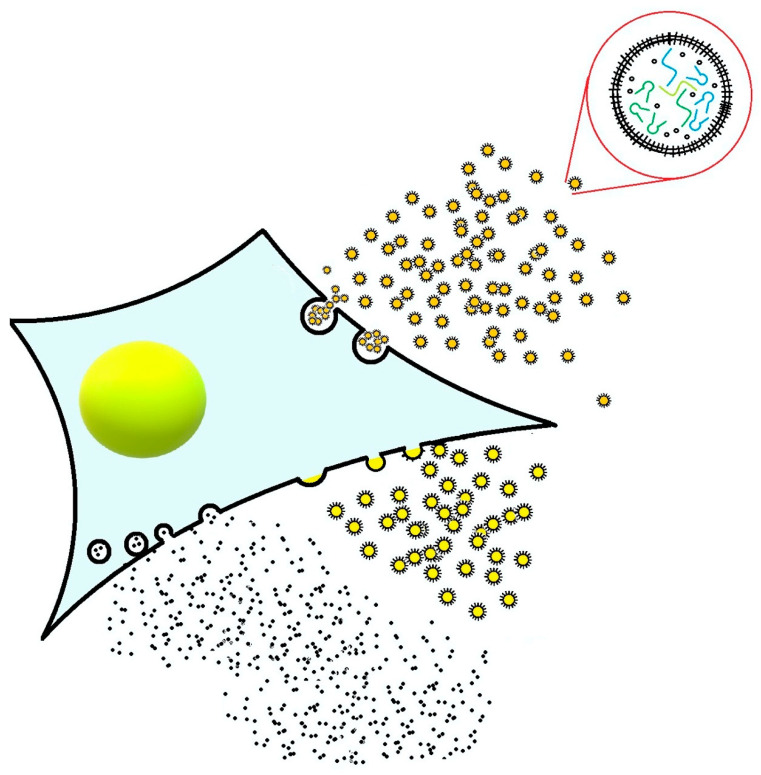
Illustration of the MSC secretome and the biogenesis of extracellular vesicles (EVs) either by direct budding from the plasma membrane or by fusion of multivesicular endosomes with the plasma membrane. The secretome is composed of soluble and vesicular (EVs) proteins, lipids, RNA (mRNA and noncoding RNAs) and DNA and influences diverse biological functions, including the immune response, endogenous cell homing and cell differentiation.

The success of current MSC therapies is affected by several factors. Age and disease state of the patient can negatively influence the proliferation and differentiation potential, morphology, and senescence of autologous MSCs and impact the therapeutic outcome [312,313,314,315]. Allogeneic MSC therapy, on the other hand, faces potential problems with both cell-mediated and humoral immune responses to MHC-mismatched allogeneic MSCs [172,173,174,316,317,318,319], which can result in adverse clinical responses and synovial inflammation following repeated intra-articular injection of allogeneic MSCs [172]. As MSCs show poor survival and engraftment at the site of injury following transplantation, they exert their therapeutic effect predominantly by secreting bioactive factors, collectively termed the “secretome” (Figure 3) [56,57,320,321,322]. The secretome is composed of soluble and vesicular (extracellular vesicles, EVs) proteins, lipids, RNA (mRNA and noncoding RNAs) and DNA and influences diverse biological functions, including the immune response, endogenous cell homing and cell differentiation [56,57,320,321,322]. The secretome mirrors the ability of the parental cells to condition and program the surrounding microenvironment, influencing a variety of endogenous responses, in injured tissues [323,324]. Secretome and EVs have shown an equivalent therapeutic potential to their parent cells in treating various conditions, including OA, graft versus host disease, myocardial ischemia/reperfusion injuries and skin wounds [325,326,327]. The MSC secretome or EVs thus have the potential to be developed into a stand-alone therapeutic product or a coadministered agent to enhance the effectiveness of cell therapy by modulating the microenvironment into a regeneration conducive milieu [328]. Indeed, recently cell-free secretome therapies have shown great potential in vitro and in pioneering clinical applications [329,330,331].

Considering the mounting economic and safety concerns over the use of exogenous cellular material [332], using paracrine factors to establish a repair-conducive microenvironment and recruit endogenous cells is a promising novel regenerative strategy. It would eliminate the need to administer exogenously manipulated cells and avoid the cost, complexity, and risk of in vitro cell expansion and reimplantation as well as the regulatory problems associated with the use of living cells [333].

Another strategy to overcome the donor-age and -health-specific therapeutic limitations of stem cells is offered by iPSC technology. The reprogramming process does not just restore pluripotency, but resets an aged, somatic cell to a more youthful state, elongating telomeres, rearranging the mitochondrial network, reducing oxidative stress and thus offers a significant therapeutic potential, although the extent to which iPSCs truly mime embryonic stem cells is controversial. However, iPSCs have been shown to harbour an epigenetic memory characteristic of their tissue of origin which may impact their differentiation potential [334].

Lastly, models of tissue injury and naturally occurring regeneration have shown the importance of the immune response for tissue repair, highlighting the necessity to modulate inflammatory processes to facilitate regeneration [335,336]. Traditional regenerative medicine focused on transplanting exogenously prepared cells or tissue while neglecting to consider the inflammatory and degenerative microenvironment [337,338]. Novel approaches try to work with, not against biology and aim to create a proregenerative milieu to induce endogenous regeneration [337,338]. To this end the genetic elements, regulatory pathways and specific cell populations that limit or allow intrinsic regeneration need to be identified to be able to use mammalian tissue development and regeneration as a blueprint to guide the development of novel regenerative therapies [335,336].

## Data Availability

The data presented in this study are available in the manuscript and Supplementary Table S1.

## Supplementary Materials

The following are available online at https://www.mdpi.com/2076-2615/11/1/234/s1, Table S1. Detailed information of all referenced in vivo studies conducted in horse or using equine cells.
