# Supplementary material for: Regenerative Medicine for Equine Musculoskeletal Diseases
Source: Animals (Basel). 2021 Jan 19;11(1):234. doi: 10.3390/ani11010234 (PMC7832834; doi:10.3390/ani11010234)
Supplement: Supplementary file 1 [file animals-11-00234-s001.pdf]

Supplementary Table S1—Detailed information of all referenced in vivo studies conducted in horses or using equine cells

Paper	Species	Number of animals in total	Presence of a control group	Pre-post intervention data present	Random selection of participants for assessment	Random assignment of participants to the intervention	Follow-up rate in %	Are the group matched according to signalment\activity level?	Major limitations mentioned in the study
**Tendon**									
Smith 2008	horses	242	no	yes	NA (not applicable)	NA	44	NA	Lacking a control group
Crovace et al. 2010	horses	6	yes	yes	yes	yes	100	no	Small number of animals, experimental model of tendon injury
Torrent et al. 2019	horses	9	no	yes	NA	NA	100	NA	
Ahrberg et al. 2018	horses	6	yes	yes	yes	yes	100	no	Using different limbs of the same animals as control and treatment groups
Carvalho et al. 2013	horses	8	yes	yes	yes	yes	100	no	Small number of animals
Lange-Consiglio et al. 2013	horses	95	no	no	NA	NA	100	NA	Lacking a control group
Castelijns et al. 2011	horses	11	no	yes	NA	NA	100	NA	
Waselau et al. 2008	horses	9	no	yes	NA	NA	100	NA	

Supplementary Table S1—Detailed information of all referenced in vivo studies conducted in horses or using equine cells

Geburek et al. 2015	horses	15	yes	yes	no	yes	100	no	Small number of animals
**Osteoarthritis**									
Nixon et al. 2015	horses	6	yes	yes	yes	yes	100	no	Multiple lesions in the same joint used as control and treatment groups
Nixon et al. 2017	horses	24	yes	no	NA	yes	100	no	Additional readouts would improve result quality
Frisbie et al. 2008	horses	15	yes	no	yes	yes	100	no	
Menarim et al. 2019	horses	6	yes	yes	yes	yes	100	no	
Frisbie et al. 2009	horses	24	yes	yes	yes	yes	100	no	
Delco et al. 2019	horses	8	yes	yes	yes	yes	100	no	
Yamada et al. 2013	horses	4	yes	yes	yes	yes	100	no	
Broeckx et al. 2019	horses	12	yes	yes	yes	yes	100	no	Small number of animals, only short term assessment
Mariñas-Pardo et al. 2018	horses	80	yes	yes	yes	yes	88	no	
Zayed et al. 2018	rats	Not mentioned	yes	yes	yes	yes	100	yes	

Supplementary Table S1—Detailed information of all referenced in vivo studies conducted in horses or using equine cells

Joswig et al. 2017	horses	18	yes	yes	yes	yes	100	no	Using normal and not diseased joints
Pezzanite et al. 2015	horses	6	yes	yes	NA	NA	100	no	Not clinically relevant injection sites were used
Pigott et al. 2013	horses	6	yes	yes	yes	yes	100	no	
Pichereau et al. 2014	horses	20	no	no	NA	NA	Not mentioned	no	
Carmona et al. 2007	horses	4	no	no	NA	NA	100	no	Small number of animals, lacking a control group
Smit et al. 2019	horses	10	yes	yes	no	no	100	no	Small number of animals, lacking a matched control group
Moraes et al. 2015	horses	8	yes	yes	yes	yes	100	no	
Textor and Tablin 2013	horses	7	yes	yes	yes	yes	100	no	
Moreira et al. 2015	horses	10	yes	yes	yes	yes	100	no	
Bertone et al. 2014	horses	20	yes	yes	yes	yes	100	no	
Tyrnenopoulou et el. 2016	horses	15	yes	yes	yes	yes	100	no	
Mirza et al. 2016	horses	12	yes	yes	no	no	100	yes	Not double-blinded, not randomized

									low numbers of participants, low number of observations, short observation period
Frisbie et al. 2007	horses	16	yes	yes	yes	yes	100	no	
Frisbie et al. 2002	horses	16	yes	yes	yes	yes	100	no	
Fjordbakk et al. 2015	horses	15	yes	NA	yes	yes	100	no	One of the ACS containers used was for human use the other was prototype container not commercially available.
**Meniscus**									
Ferris et al. 2012	mice	12	yes	yes	yes	yes	100	yes	Small number of animals
González-Fernández et al. 2016	horses	6	yes	yes	yes	yes	100	yes	Small number of animals
**Bone**									
Golafshan et al. 2020	ponies	8	yes	yes	yes	yes	100	yes	
Perrier et al. 2008	horses	9	yes	yes	yes	yes	100	no	experimental model of bone fracture
Kawcak et al. 2000	horses	8	yes	yes	yes	yes	100	no	

Supplementary Table S1—Detailed information of all referenced in vivo studies conducted in horses or using equine cells

Ishihara et al. 2010	horses	6	yes	yes	yes	yes	100	no	
Seo et al. 2015	horses	5	yes	yes	yes	yes	100	no	No clinical evaluation
McDuffee et al. 2012	horses	10	yes	yes	yes	yes	100	no	experimental model of bone fracture
McDuffee et al. 2014	mice	13	yes	yes	yes	yes	100	yes	
Seo et al. 2014	horses	7	yes	yes	yes	yes	100	no	
**Laminitis**									
Angelone et al. 2017	horses	9	no	yes	NA	NA	100	no	Small number of animals
